# Preoperative Radiographic Prediction Tool for Early Postoperative Segmental and Lumbar Lordosis Alignment After Transforaminal Lumbar Interbody Fusion

**DOI:** 10.7759/cureus.18175

**Published:** 2021-09-21

**Authors:** Ken Porche, Alexander Dru, Rachel Moor, Paul Kubilis, Sasha Vaziri, Daniel J Hoh

**Affiliations:** 1 Neurosurgery, University of Florida, Gainesville, USA; 2 Neurosurgery, University of Florida College of Medicine, Gainesville, USA

**Keywords:** transforaminal lumbar interbody fusion, radiographic outcomes, posterior lumbar interbody fusion, lumbar spine fusion, lordosis

## Abstract

Objective

Transforaminal lumbar interbody fusion (TLIF) is a common approach and results in varying degrees of lordosis correction. The purpose of this study is to determine preoperative radiographic spinopelvic parameters that predict change in postoperative segmental and lumbar lordosis after TLIF.

Materials & Methods

This study is a single surgeon retrospective review of one-level and two-level TLIFs from L3-S1. All patients underwent bilateral facetectomies, 10 mm TLIF cage (non-lordotic) insertions, and bilateral pedicle screw-rod construct placements. Pre- and post-operative X-rays were assessed for preoperative segmental lordosis (SL), lumbar lordosis (LL), and pelvic incidence (PI). Univariate and multi-predictor linear regression analyses were performed to determine the relationships between preoperative radiographic findings and change in early postoperative segmental and lumbar lordosis.

Results

Ninety-seven patients contributing 128 intervertebral segments were examined. The mean change in SL after TLIF was 7.3 (range: 0.10-28.9°, SD 6.39°). The mean change in LL after TLIF was 5.5˚ (range: -14.8-39.2°, standard deviation (SD) 7.16°). Greater preoperative LL predicted less postoperative LL correction, while greater preoperative PI predicted more postoperative SL and LL correction. Greater anterior disk height was noted to be associated with a decreased change in SL (∆SL). An annular tear on preoperative magnetic resonance imaging (MRI) predicted a 2.7° decrease in ∆SL. A Schmorl's node on preoperative MRI predicted a 4.0° decrease in change in LL (∆LL).

Conclusions

A greater preoperative lordosis and a lower spinopelvic mismatch lessen the potential for an increase in the postoperative SL and LL after a TLIF, which is likely due to a ‘ceiling’ effect of an otherwise optimized spinal alignment. A greater anterior disk height and the presence of an annular tear are associated with decreased ∆SL.

## Introduction

Transforaminal lumbar interbody fusion (TLIF) is a widely used, safe, and efficacious approach for surgical management of lumbar degenerative disease and spinal deformity [1-4]. TLIF is commonly performed to restore disk height, achieve circumferential neural decompression, relieve neuroforaminal stenosis, and augment posterior construct rigidity [5, 6]. A unique potential advantage of lumbar interbody fusion is restoration of segmental lordosis (SL) and optimization of sagittal alignment [7-11]. When compared to stand-alone posterior fusion, the reduction of pelvic incidence (PI)-lumbar lordosis (LL) mismatch achieved with TLIF results in decreased adjacent segment disease, diminished need for revision surgery, and improved postoperative patient satisfaction [12, 13].

Sagittal imbalance is the primary predictor of disability in adult spinal deformity, and restoration of sagittal alignment with spinopelvic harmony (or matched PI and LL) is known to lead to the best long-term outcomes in patients who undergo surgical correction [14, 15, 16, 17]. Previous studies have cited varying degrees of lordosis correction following TLIFs. These widely range from 2.1 - 27.3 degrees, depending on factors such as the use of lordotic cages, the number of levels instrumented, and whether the TLIF was performed in an open versus minimally invasive fashion [8, 9, 18-29]. Traditionally, radiographic factors, such as bridging osteophytes or the presence of intradiscal vacuum phenomenon, have been used to predict the capacity to restore SL [30]. Additional factors cited in the literature that may contribute to greater lordosis restoration include multilevel fusion, cage size, use of the cantilever TLIF technique, low preoperative SL, and high preoperative spinopelvic mismatch [18, 19, 24, 31, 32].

Currently, there are limited studies that use patient-specific imaging characteristics to predict SL correction after TLIF [33]. Therefore, the objective of this analysis is to demonstrate a patient-specific algorithm for the prediction of early postoperative degree of SL and LL correction after TLIF.

## Materials and methods

After obtaining approval from the University of Florida's internal review board (IRB202002814), we conducted a single surgeon retrospective review of all patients that underwent one- and two-level TLIFs from levels L3-S1, between 2010 and 2015. Data was de-identified and therefore, informed consent was not sought. Patients less than 18 years of age were excluded from this study. Each patient included in the study had preoperative 36-inch standing x-rays and post-operative lumbar x-rays. Additionally, 39% had a preoperative lumbar computerized tomography (CT) scan and 95% had a preoperative lumbar MRI. Each patient included in the study underwent a standard open TLIF technique (as previously described) involving a laminectomy with complete bilateral facetectomies, bilateral pedicle screw-rod construct placements, and placement of a 10mm non-lordotic cage (Concorde, Depuy Synthes, Raynham, MA, USA).

Data acquisition

Preoperative and immediate postoperative x-rays were assessed for spinopelvic parameters, including LL, SL and PI. LL was defined as the Cobb angle between the superior endplates of L1 and S1. SL was defined as the Cobb angle between the superior endplate above and the inferior endplate below the operative level. PI was defined as the angle between a line perpendicular to the sacral plate at its midpoint and a line connecting this point to the femoral head axis. The spinopelvic mismatch was calculated as PI-LL. Changes in LL and SL were calculated by determining the difference between pre- and postoperative Cobb angles of the lumbar spine and at the level(s) of operation, respectively. Immediate postoperative x-rays were used to determine the change in the SL and LL to reflect the primary effect of the TLIF procedure on spinal alignment. Additional radiographic assessments of the intervertebral segment(s) at the operative level(s) were performed using preoperative MRI and CT scans, including the anterior disk height, annular tears, presence of a Schmorl's node, Modic type endplate change, bridging osteophytes, and vacuum disk. All preoperative disk characteristics and pre-/postoperative spinal measurements were performed and collected by two individuals (KP and AD), with a calculated discordance of 2.3% when considering all values included in the study.

Statistical Methods

We used the mixed effect linear regression to fit single- and multi-predictor models of change in LL (ΔLL) and SL (ΔSL) [34, 35]. Possible non-linearity of continuous predictors was evaluated using restricted cubic splines and co-linearity was evaluated using a variable clustering method and variance inflation factors (VIF) cutoff of 2 [34]. We then applied a backward elimination selection procedure to the remaining predictors [34]. We re-ran the backward elimination process on 100 bootstrap samples of the original dataset and tallied the frequency with which each predictor from our original candidate list was selected over the 100 final model fits. Our final best models consisted of predictors that were selected in > 50% of these model fits.

We used the adjusted R-square to assess the predictive performance of our best multi-predictor linear regression models and validated using Efron's "optimism" [34]. Residual analysis indicated normally distributed errors for both outcome models. Best model regression coefficients are presented along with 95% confidence limits and t statistic p-values for tests of whether the coefficients differed significantly from zero. We identified the region of the ΔLL and ΔSL response surfaces defined by PI and preoperative LL that differed significantly from ΔLL=0 or ΔSL=0, adjusting the confidence region using Scheffe's adjustment for multiple testing [34]. Statistical calculations were performed using SAS Version 9.4 (SAS Institute, Cary NC) and R Version 3.5.0 (R Foundation for Statistical Computing, Vienna, Austria).

## Results

A total of 97 patients undergoing TLIFs at 128 levels were examined (Table 1). Sixty-six patients underwent single level, and 31 two-level TLIFs. The mean age was 62.5 years (standard deviation (SD) 11.63), and 44 (47.3%) of the patients were male, with an average body mass index (BMI) of 30.24 (SD 6.14). The LL analysis consisted of 66 one-level patients with L3-L4, L4-L5, and L5-S1 disk involvements and 27 two-level patients. The SL analysis consisted of 66 disks from one-level patients and 54 disks from two-level patients.

**Table 1:** Frequencies and percent occurrences of patient and disk space characteristics. *Note: 1 missing for each of these variables due to missing magnetic resonance imaging.

Variable		N (%)
All patients		93
All disks		120
1-Level		66 (71.0)
	L3-L4	17 (18.3)
	L4-L5	34 (36.6)
	L5-S1	15 (16.1)
2-Level		27 (29.0)
	L3-L5	7 (7.5)
	L3-L4 & L5-S1	1 (1.1)
	L4-S1	19 (20.4)
Male		44 (47.3)
Female		49 (52.7)
Prior surgery		39 (41.9)
Bridging osteophytes		10 (10.8)
Vacuum disks		51 (54.8)
Annular tears		28 (31.1)*
Modic changes		48 (53.3)*
Schmorl's nodes		19 (21.4)*

Preoperative intervertebral segment characteristics

The average anterior disk height was 8.41mm (range: 0.14-23.20, SD 3.85). There were 32 patients with annular tears (26.02%), 26 patients with a Schmorl's node (21.31%), 63 patients with Modic type endplate changes (51.22%), and 60 patients with intradiskal vacuum phenomena (46.88%).

Preoperative spinopelvic parameters

The average preoperative PI was 56.96 (SD 14.12) (Table 2). The average preoperative LL was 48.79 (SD 11.47), and the average preoperative SL was 16.64 (SD 8.16). The mean preoperative PI-LL mismatch was 8.05 (SD 13.80).

**Table 2:** Means and standard deviations (SD) of patient and disk space characteristics. N = number, SD = standard deviation; BMI = body mass index; SL = segmental lordosis; PI = pelvic incidence; LL = lumbar lordosis; change in SL (∆SL); change in LL (∆LL).

Variable	N	Mean ± SD
Age	93	62.25 ± 11.53 yrs
BMI	93	30.02 ± 6.12
Anterior disk height	120	8.42 ± 3.93 mm
Preop SL	120	16.64 ± 8.16°
PI	93	56.96 ± 14.12°
Preop LL	93	48.91 ± 11.10°
PI-LL	93	8.05 ± 13.80°
Postop SL	120	23.97 ± 8.51°
Postop LL	93	54.40 ± 10.86°
ΔSL	120	7.33 ± 6.39°
ΔLL	93	5.50 ± 7.16°

Immediate postoperative change in segmental and lumbar lordoses

All TLIF levels demonstrated an increase in SL (ΔSL), with a mean improvement of 7.33° (range: 0.10-28.9°, SD 6.39°). Univariate analysis revealed that greater preoperative SL negatively impacted postoperative ΔSL (p<0.01, 95% CI -0.40° to -0.12°) (Table 3). Additionally, a greater anterior disk height correlated with a significantly decreased postoperative ΔSL (p=0.02, 95% CI -0.66° to -0.06°). For each 1 mm increase in preoperative anterior disc height, there was a 2.8° lowering in postoperative improvement in SL. A greater preoperative PI-LL mismatch was significantly associated with an increased postoperative ΔSL (p=0.03, 95% CI 0.01° to 0.19°). For each 10° increase in preoperative PI-LL mismatch, there was a 1.0° increase in postoperative SL.

**Table 3:** Univariate mixed-effect linear regression coefficients for predictors of change in segmental lordosis. *Note: comparison was made with the L5-S1 disk space. SE = Standard Error; CI = Confidence Interval; BMI = body mass index; Preop = preoperative; SL = segmental lordosis; PI = pelvic incidence; LL = lumbar lordosis

Effect		B (°)	SE (°)	95% CI (°)		p-value
				Lower	Upper	
Level (2- vs 1-level)		-1.41	1.27	-4.01	1.20	0.2785
Disk space						0.8576
	L3-L4*	0.90	1.65	-2.51	4.30	0.5915
	L4-L5*	0.28	1.27	-2.39	2.85	0.8593
Age		0.05	0.05	-0.06	0.15	0.3859
BMI		0.04	0.10	-0.17	0.25	0.6888
Anterior disc height (mm)		-0.36	0.14	-0.66	-0.06	0.0197
Sex (male compared to female)		-0.01	1.25	-2.57	2.55	0.9931
Prior surgery		-0.55	1.25	-3.12	2.03	0.6663
Bridging osteophytes		1.60	2.04	-2.58	5.79	0.4383
Vacuum disk		0.57	1.15	-1.79	2.93	0.6244
Annular tears		-2.41	1.32	-5.12	0.31	0.0797
Modic changes		0.19	1.19	-2.24	2.63	0.8716
Schmorl's nodes		0.73	1.52	-2.39	3.86	0.6341
Preop SL		-0.26	0.07	-0.40	-0.12	0.0009
PI		0.07	0.04	-0.02	0.16	0.1326
Preop LL		-0.04	0.06	-0.15	0.07	0.4786
PI-LL		0.10	0.05	0.01	0.19	0.0303

**Figure 1:**
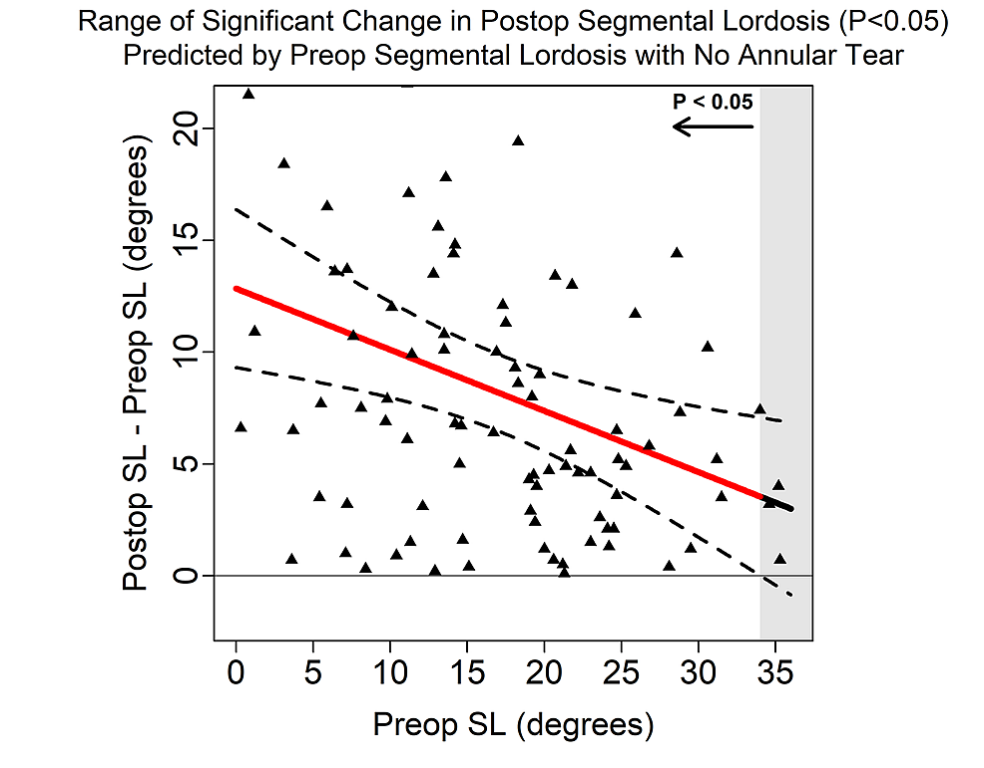
95% Scheffe confidence band which demonstrates that the change in segmental lordosis (SL) is significant if the preoperative SL is <34° when no annular tear is found within that segmental level. A 4° decrease in the preoperative SL predicted a single degree greater change in the SL correction. Postop = Postoperative; SL = Segmental Lordosis; Preop = Preoperative

**Figure 2:**
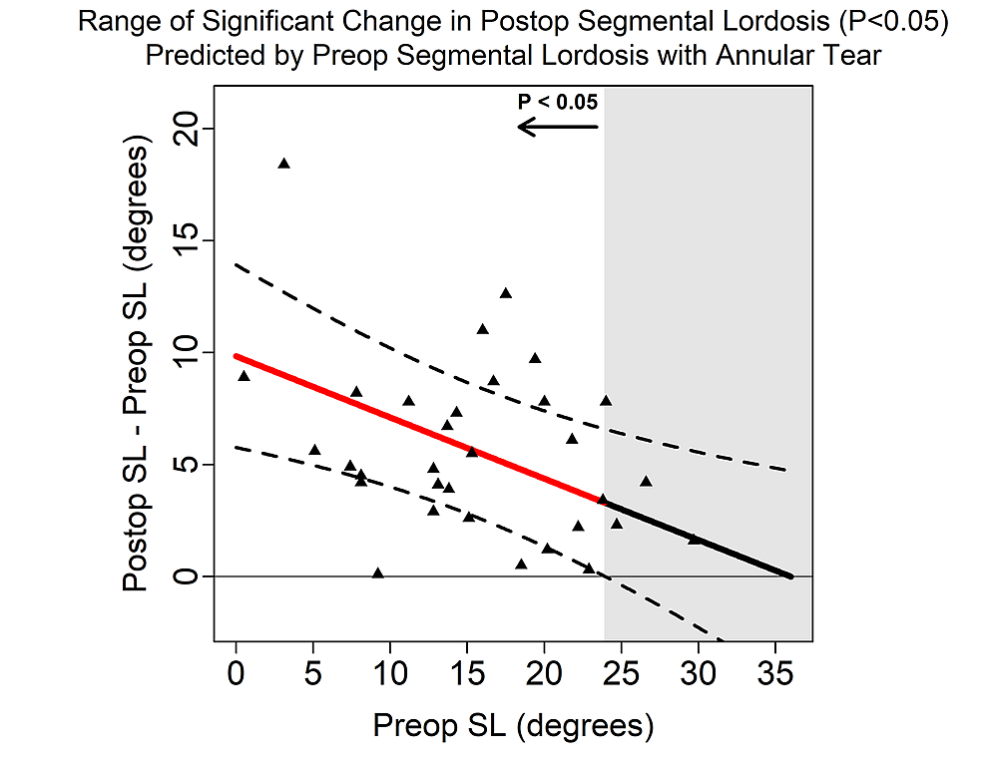
The above represents a 95% Scheffe confidence band which demonstrates that a change in segmental lordosis (SL) is significant if the preoperative SL is <23.9° when an annular tear is found within that segmental level. A 4° decrease in the preoperative SL predicted a single degree more SL correction. Additionally, an annular tear predicted 2.4° less correction. Postop = Postoperative; SL = Segmental Lordosis; Preop = Preoperative

**Figure 3:**
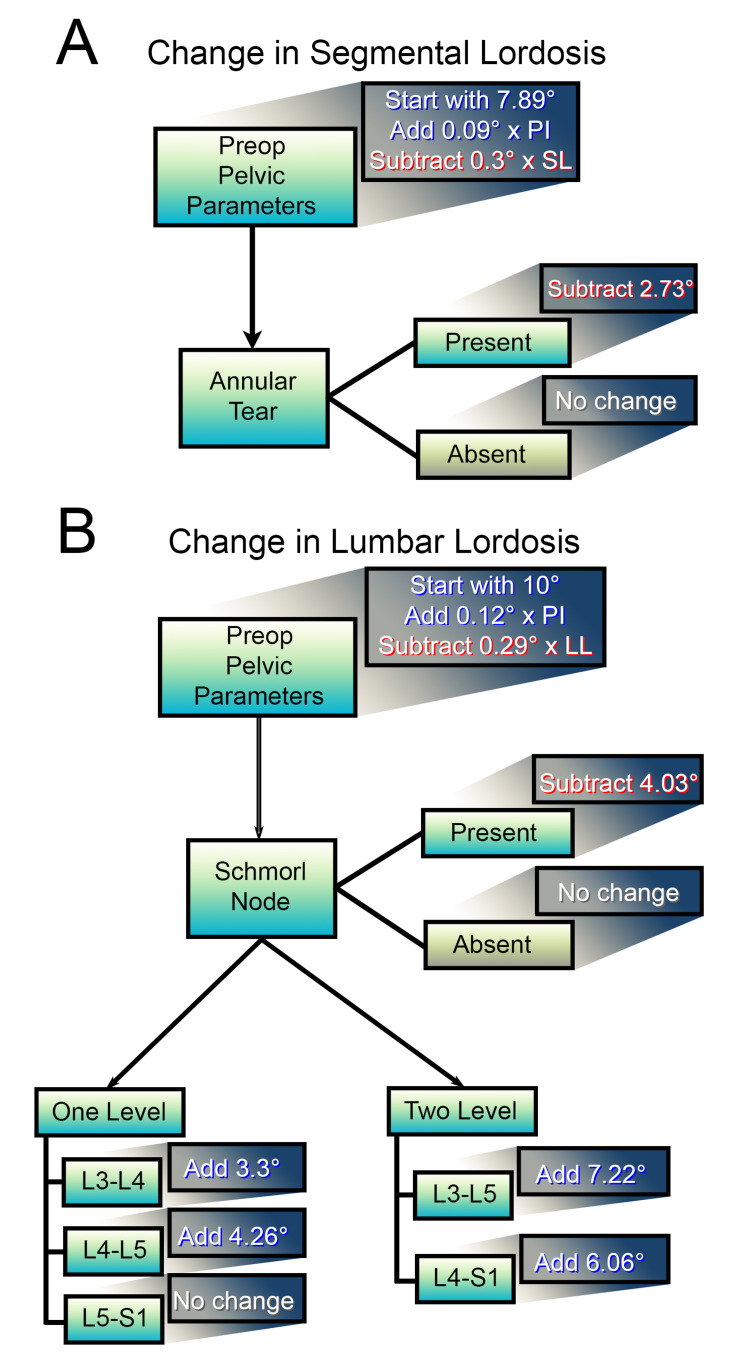
Flowchart for (A) predicting change in the segmental lordosis (ΔSL) where (ΔSL) = 7.89° - 0.3 [Preop SL]° + 0.09 [Preop PI]° - 2.73°ᵃ [note: subtract 2.73° only if an annular tear is present] and (B) predicting change in the lumbar lordosis (ΔLL) after one- and two-level transforaminal lumbar interbody fusions (TLIFs) where ΔLL= 10° + 0.12 [Preop PI]° − 0.29 [Preop LL]° + [Level(s)ᵃ]° - 2.73°ᵇ where a for L3-L4 = 3.30, L4−L5 = 4.26, L3−L5 = 7.22, L4−S1 = 6.06 and for L5−S1 = 0 and b is subtracted only if a Schmorl's node is present. The blue highlight indicates added degrees and the red highlight indicates subtracted degrees. At most one addition should be made for each operative level; however, no change is made for operative level for one-level L5-S1 TLIFs. For example, a single level L4-5 TLIF with a preoperative SL of 7.8°, a preoperative LL of 43.7°, and a PI of 65.0°, when an annular tear is present but no Schmorl's node is present, the ΔSL is predicted to be 8.6° [i.e. 7.89° + 0.09(65°) - 0.3(7.8°) - 2.73° = 8.6°] and the ΔLL is predicted to be 9.4° [i.e. 10° + 0.12(65°) - 0.29(43.7°) - 0° + 4.26° = 9.4°]. $\Delta SL= 7.89 ^{\circ}-0.3\left [ Preop SL \right ]^{\circ}+0.09\left [Preop PI \right ]^{\circ}-2.73^{\circ a} \\ a: subtract\:only\:if\:annular\:tear\:present$ $\Delta LL= 10^{\circ}+0.12\left [Preop PI \right ]^{\circ}-0.29\left [ Preop LL \right ]^{\circ}+\left [ Level(s)^{a} \right ]^{\circ}-2.73^{\circ b} \\ a: L3-L4=3.30,\:L4-L5=4.26,\:L3-L5=7.22,\:L4-S1=6.06,\:L5-S1=0\\ b: subtract\:only\:if\:Schmorl's\:node\:present$ SL = Segmental lordosis; PI = Pelvic incidence; LL = Lumbar lordosis

Using multi-predictor linear regression models of ΔSL, the presence of an annular tear was significantly associated with less ΔSL (p=0.02, 95% confidence interval (CI) -5.34° to -0.38°) (Table 4). The presence of an annular tear decreased the ∆SL by 3°. The ΔSL in patients without an annular tear was statistically significant (p<0.05) for values of preoperative SL < 34° (Figure 1). ΔSL in patients with an annular tear was statistically significant (p<0.05) for values of preoperative SL < 23.9° (Figure 2). Preoperative LL was not found to be associated with significant ΔSL (p=0.54). An equation for ΔSL prediction based on significant factors is demonstrated below; see associated flowchart (Figure 3A). 

**Table 4:** Multi-predictor linear regression model of change in segmental lordosis. Validated estimate of adjusted R-squared of 0.1449. SE = standard error; CI = Confidence Interval; Preop = Preoperative; PI = Pelvic incidence; LL = lumbar lordosis

Effect	B (°)	SE (°)	95% CI (°)		p-value
			Lower	Upper	
Intercept	7.19	2.64	1.96	12.41	0.0074
Preop SL	-0.32	0.08	-0.48	-0.17	<0.0001
PI	0.08	0.04	-0.01	0.16	0.0721
Preop LL	0.04	0.06	-0.09	0.16	0.5452
Annular Tear	-2.86	1.25	-5.34	-0.38	0.0242

The SL cohort was further stratified into the 5th percentile (3.4°), the mean (16.6°), and the 95th percentile (30.9°). This analysis demonstrated that for a mean preoperative SL with an annular tear, a statistically significant ΔSL was only obtained if the preoperative LL was >35° and the PI was >31°. With a preoperative SL in the 95th percentile, but without an annular tear, a statistically significant ΔSL was only obtained if the preoperative LL was >50° and the PI was >60°. No significant change was observed for those with a preoperative SL in the 95th percentile with an annular tear. Otherwise, all other preoperative spinopelvic parameters were observed to cause significant post-operative SLs.

**Figure 4:**
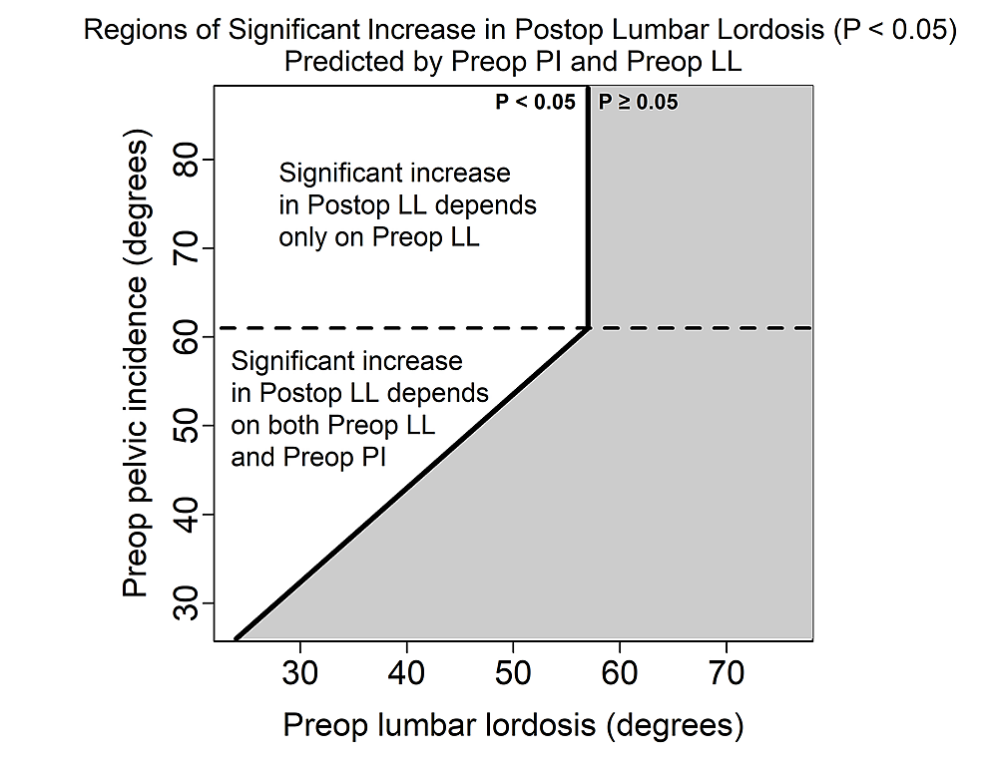
Representative plot demonstrating regions where preoperative lumbar lordosis (LL) and pelvic incidence (PI) predict significant correction in LL. A significant ΔLL was observed when PI was greater than preoperative LL. A “ceiling” effect is observed at a LL of 57° which matches the average PI of 57°.

All TLIF levels demonstrated improved ΔLL, with a mean change of 5.50° (range: -14.8-39.2°, SD 7.16°). Univariate analysis revealed a 3.18° increase in LL correction with two-level over one-level surgeries (Table 5). In both the univariate analysis and the multi-predictor linear regression model of ΔLL, greater preoperative LL negatively impacted ΔLL (multi-predictor: p<0.01, 95% CI -0.43° to -0.15°) while increases in PI significantly increased ΔLL (multi-predictor: p=0.03, 95% CI 0.01° to 0.23°) (Table 6). A 3° increase in preoperative LL predicted a single degree decrease in ΔLL, while an 8° increase in PI predicted a single degree increase in ΔLL. The presence of a Schmorl's node predicted a 4.03° decrease in ΔLL (p=0.03, 95% CI -7.62° to -0.43°). A significant ΔLL was observed when PI was > preoperative LL as long as preoperative LL was < 57° (Figure 4). An equation for ΔLL prediction based on significant factors is demonstrated below; see associated flowchart (Figure 3B).

**Table 5:** Univariate linear regression coefficients for predictors of change in lumbar lordosis. *Note: comparison was made with the L5-S1 disk space. SE = Standard Error; CI = Confidence Interval; BMI = Body mass index; Preop = Preoperative; PI = Pelvic incidence; LL = lumbar lordosis

Effect		B (°)	SE (°)	95% CI (°)		p-value
				Lower	Upper	
Level (2- vs 1-level)		3.18	1.61	-0.02	6.37	0.0515
Disk space *						0.0894
	L3-L4	5.42	2.47	0.51	10.34	0.0310
	L4-L5	3.84	2.16	-0.46	8.13	0.0793
	L3-L5	8.05	3.19	1.71	14.39	0.0135
	L3-L4 & L5-S1	1.89	7.20	-12.42	16.21	0.7933
	L4-S1	6.24	2.41	1.45	11.03	0.0112
Age		0.12	0.06	-0.00	0.25	0.0593
BMI		0.04	0.12	-0.21	0.28	0.7722
Sex (male compared to female)		-0.55	1.49	-3.52	2.41	0.7114
Prior surgery		1.50	1.50	-1.49	4.49	0.3214
Bridging osteophytes		2.71	2.39	-2.04	7.46	0.2601
Vacuum disks		-0.15	1.50	-3.13	2.83	0.9205
Annular tears		1.08	1.63	-2.17	4.33	0.5101
Modic changes		-1.88	1.51	-4.88	1.11	0.2150
Schmorl's nodes		-2.38	1.84	-6.05	1.28	0.1998
PI		0.01	0.05	-0.10	0.12	0.8563
Preop LL		-0.23	0.06	-0.36	-0.11	0.0004
PI-LL		0.16	0.05	0.06	0.26	0.0026

**Table 6:** Multi-predictor linear regression model of change in lumbar lordosis. Validated estimate of adjusted R-squared of 0.1547. *Note: comparison was made with the L5-S1 disk space. SE = Standard Error; CI = Confidence Interval; PI = Pelvic incidence; Preop = Preoperative; LL = lumbar lordosis

Effect		B (°)	SE (°)	95% CI (°)		p-value
				Lower	Upper	
Intercept		9.99	3.79	2.45	17.52	0.0100
PI		0.12	0.05	-0.01	0.23	0.0290
Preop LL		-0.29	0.07	-0.43	-0.15	<0.0001
Disk space *						0.0904
	L3-L4	3.30	2.30	-1.27	7.88	0.1550
	L4-L5	4.26	2.03	0.23	8.29	0.0388
	L3-L5	7.22	2.99	1.28	13.17	0.0179
	L3-L4 & L5-S1	2.62	6.55	-10.40	15.64	0.6898
	L4-S1	6.06	2.20	1.68	10.43	0.0072
Schmorl's nodes		-4.03	1.81	-7.63	-0.43	0.0287

## Discussion

All patients in our study had improvement of segmental lordosis only in the immediate postoperative period, with variation based on characteristics of the intervertebral segment and preoperative spinopelvic parameters. Of the intervertebral segment characteristics we studied, anterior disk height and the presence of an annular tear at least marginally impacted postoperative segmental lordosis change. Patients with a higher preoperative anterior disk height had significantly less ∆SL for a given instrumented level (i.e., there was a 2.8° reduction with each additional 1mm of anterior height). This suggests a decreased capacity for lordosis correction in these patients, which is a “ceiling" effect as these patients may already have maximal, well-preserved segmental lordosis. This effect, however, lost statistical significance when all predictors were considered, likely due to high variability. The presence of an annular tear was associated with a less postoperative change in SL (2.9°). This may be explained by the association of an annular tear with a more severely degenerated and less mobile disk. Alternatively, the small effect size of < 3 degrees may not represent a true clinically relevant finding. 

Lower preoperative SL and high preoperative spinopelvic mismatch are both associated in the literature with greater postoperative lordosis restoration [18,19,24]. Our study also found that a smaller preoperative SL and greater spinopelvic mismatch significantly increased postoperative ∆SL and ∆LL. Patients with a lower preoperative SL and LL, and a high mismatch logically have more capacity for restoration of lordosis, and this was demonstrated in our study. It also may suggest that improvement in SL and LL were a primary indication for surgery in these patients, which may have introduced selection bias. It also appears that lordosis restoration depends on both preoperative LL and PI when preoperative LL is < approximately 57°, which correspondingly was the average PI in this study population (i.e. when the LL is low enough to create a mismatch with the PI). When the preoperative LL is >57°, postoperative LL appears to depend only on PI. This may suggest a “ceiling" effect in which an individual with an already optimized preoperative LL cannot achieve additional lordosis correction by undergoing a TLIF. This “ceiling” is dependent on the level of operation and disk characteristics; it decreases with more rostral levels and with the presence of Schmorl's nodes. While Schmorl's nodes had no effect on segmental lordosis, they predicted a 4° decrease in achievable lordotic correction, which may be due to the more global degenerative disease/rigidity that they represent.

This study has several limitations including its retrospective design and inclusion of patients from a single surgeon and a standardized technique for open TLIF, which may impact the generalizability of the findings. Postoperative radiographic assessment was performed using immediate postoperative x-rays, and not at longer-term follow-up. This study was designed to isolate the primary effect of the TLIF procedure on change in LL and SL. It is well understood that delayed subsidence may occur over time which may reduce LL and SL, but this is likely variably affected by individual patient factors (e.g. bone mineral density and BMI). Therefore, the study findings observed here likely represent the maximal extent of lordosis correction at the index surgical procedure and may demonstrate loss of effect in some patients over time. Additionally, our prediction equations require additional power in order to further reduce error.

## Conclusions

The TLIF is a valuable procedure for restoration of lordosis, but individualized intervertebral segment and spinal alignment parameters may limit the degree of attainable correction. A greater anterior disk height and an annular tear are associated with a decreased ∆SL. Greater initial lordotic angles and lower spinopelvic mismatch lessen the potential for change in LL and SL due to a ‘ceiling’ effect of an otherwise well-aligned spine. Alternatively, patients with significant preoperative losses of segmental and overall lumbar lordosis may demonstrate potentially greater corrections of spinal alignment with TLIFs.
